# A Systematic Review and Meta-Analysis on the Management and Outcome of Isolated Skull Fractures in Pediatric Patients

**DOI:** 10.3390/children10121913

**Published:** 2023-12-12

**Authors:** Lucca B. Palavani, Raphael Bertani, Leonardo de Barros Oliveira, Sávio Batista, Gabriel Verly, Filipi Fim Andreão, Marcio Yuri Ferreira, Wellingson Silva Paiva

**Affiliations:** 1 Faculty of Medicine, Max Planck University Center, Indaiatuba 13343-060, Brazil; lucca.palavani730@al.unieduk.com.br; 2 Faculty of Medicine, São Paulo University, São Paulo 05508-220, Brazil; 3 Faculty of Medicine, State University of Ponta Grossa, Ponta Grossa 84010-330, Brazil; 4 Faculty of Medicine, Federal University of Rio de Janeiro, Rio de Janeiro 21941-617, Brazil; saviobatista@ufrj.br (S.B.); gabrielverly@ufrj.br (G.V.);; 5 Faculty of Medicine, Ninth July University, São Paulo 02117-010, Brazil

**Keywords:** isolated skull fracture in children, pediatric traumatic brain injury, pediatric

## Abstract

Background: The impact of traumatic brain injury (TBI) on the pediatric population is profound. The aim of this study is to unveil the state of the evidence concerning acute neurosurgical intervention, hospitalizations after injury, and neuroimaging in isolated skull fractures (ISF). Materials and Methods: This systematic review was conducted in accordance with PRISMA guidelines. PubMed, Cochrane, Web of Science, and Embase were searched for papers until April 2023. Only ISF cases diagnosed via computed tomography were considered. Results: A total of 10,350 skull fractures from 25 studies were included, of which 7228 were ISF. For the need of acute neurosurgical intervention, the meta-analysis showed a risk of 0% (95% CI: 0–0%). For hospitalization after injury the calculated risk was 78% (95% CI: 66–89%). Finally, for the requirement of repeated neuroimaging the analysis revealed a rate of 7% (95% CI: 0–15%). No deaths were reported in any of the 25 studies. Conclusions: Out of 7228 children with ISF, an almost negligible number required immediate neurosurgical interventions, yet a significant 74% were hospitalized for up to 72 h. Notably, the mortality was zero, and repeat neuroimaging was uncommon. This research is crucial in shedding light on the outcomes and implications of pediatric TBIs concerning ISFs.

## 1. Introduction

The impact of traumatic brain injury (TBI) on the pediatric population is profound, as it stands as a leading cause of both fatalities and disabilities [1,2]. While TBI’s severity in adults is widely acknowledged, the unique pathophysiological aspects involved in pediatric cases magnify the associated burden, making it even more substantial [1].

Pediatric brain injuries present distinctive biomechanical characteristics due to the heightened plasticity and deformability of the developing brain. The infant skull, less rigid and featuring flexible sutures that act like joints, allows for some movement in response to mechanical stress, potentially resulting in birth-related injuries such as intracranial hemorrhages caused by compression and traction during delivery [3,4]. Additionally, shaking can cause a slight deformity of the skull and redistribute forces within, potentially leading to stretching and shearing injuries. Furthermore, children’s relatively larger heads make them more vulnerable to head trauma compared to adults [1,5].

An isolated skull fracture (ISF) stands as a distinct focal point within the complex spectrum of TBI. TBI, in its broad context, is associated with severe and multifaceted consequences. However, the ISF introduces its own set of distinctive characteristics that warrant specific attention. Extensive analysis, through prior comprehensive meta-analyses, has diligently explored the short-term implications of this condition. The collective findings from these studies ultimately revealed a rather low likelihood of emergent neurosurgical intervention or fatality in cases of ISF. Despite this relatively low risk, it is noteworthy that ISF cases tend to exhibit a significantly high incidence of hospitalization. This suggests that although the immediate life-threatening aspect is relatively rare, the injury itself necessitates a considerable degree of medical care and observation due to its potentially severe nature, ultimately concluding that the risk of emergency neurosurgery or fatality is exceedingly low, yet it is accompanied by a notably high rate of hospitalization [6].

Despite the establishment of guidelines for TBI in children [7,8], there is room for research, as new evidence of ISF in the pediatric population has emerged since the publication of the last meta-analysis [6]. In this matter particularly, there is no robust current evidence demonstrating the requirement of acute neurosurgical intervention, hospitalizations after injury, and neuroimaging in ISF [9]. Hence, the authors conducted a single-arm update meta-analysis to unveil the state of the evidence concerning these factors.

## 2. Materials and Methods

### 2.1. Eligibility Criteria

Inclusion in this meta-analysis was restricted to studies that met all the following criteria (1) randomized or non-randomized studies; (2) report isolated pediatric skull fracture on computed tomography (CT) scan; (3) studies that report one of the interest outcomes; (4) studies reporting four or more patients. We excluded non-English papers, reviews, letters to the editor, abstracts, and commentaries from the initial assessment.

### 2.2. Search Strategy

We systematically searched for isolated pediatric skull fractures on PubMed, Cochrane, Web of Science, and Embase databases with the following terms: (Pediatric OR child) AND (“skull fracture” OR “head injury” OR “head trauma”) AND (“surgical intervention” OR “neurosurgical intervention” OR “surgical treatment”) AND (“conservative care” OR management OR “conservative management”). Due to the lack of randomized controlled trials, our sample is mostly composed of non-randomized studies. The references from all included studies, previous systematic reviews, and meta-analyses were also searched manually for any additional studies. Two authors (F.A. and L.B.P.) independently extracted the data following predefined search criteria.

### 2.3. Outcomes Definitions

Main outcomes were defined considering mortality, the necessity of acute neurosurgery intervention, repeated neuroimaging, and hospitalizations. Cases of ISFs were considered only if they were diagnosed through CT scanning.

### 2.4. Quality Assessment

Two authors (F.A and L.B.P) independently evaluated the study quality, and any differences in their assessments were resolved via consensus. ROBINS-I scale was employed to assess the studies. By utilizing this standardized assessment tool, our objective was to assess the methodological rigor and quality of the studies included in our analysis.

### 2.5. Statistical Analysis

This systematic review and meta-analysis were performed following the Cochrane Collaboration and the Preferred Reporting Items for Systematic Reviews and Meta-Analysis (PRISMA) statement guidelines [10]. Relative risk (RR) with 95% confidence intervals was used to compare outcomes in specific treatment scenarios. Cochran Q test and I^2^ statistics were used to assess for heterogeneity; *p*-value inferior to 0.05 and I^2^ < 35% were considered significant for heterogeneity. Review Manager was used for statistical analysis.

## 3. Results

### 3.1. Study Selection

**Figure 1:**
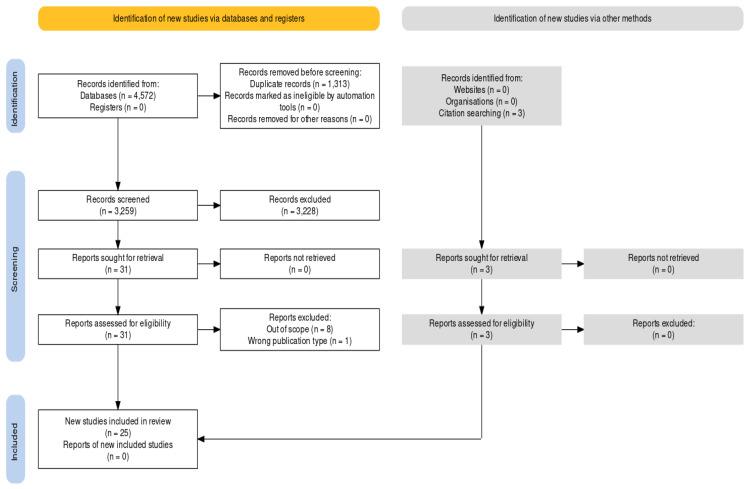
Prisma Flow Diagram.

We located a total of 4572 articles through our search efforts, with 1935 found in PubMed, 1616 in Embase, 994 in Web of Science, and 27 in Cochrane database. After the initial screening, where we assessed 3259 non-duplicate citations, we excluded 3228 articles based on title or abstract screening, leaving us with 31 articles for a full-text review. Subsequently, nine articles were excluded during the full-text screening and data extraction process. Next, three citations were manually added. Ultimately, we included 25 studies in our final analysis [9,11,12,13,14,15,16,17,18,19,20,21,22,23,24,25,26,27,28,29,30,31,32,33,34], as outlined in Figure 1.

### 3.2. Quality Assessment

**Figure 2:**
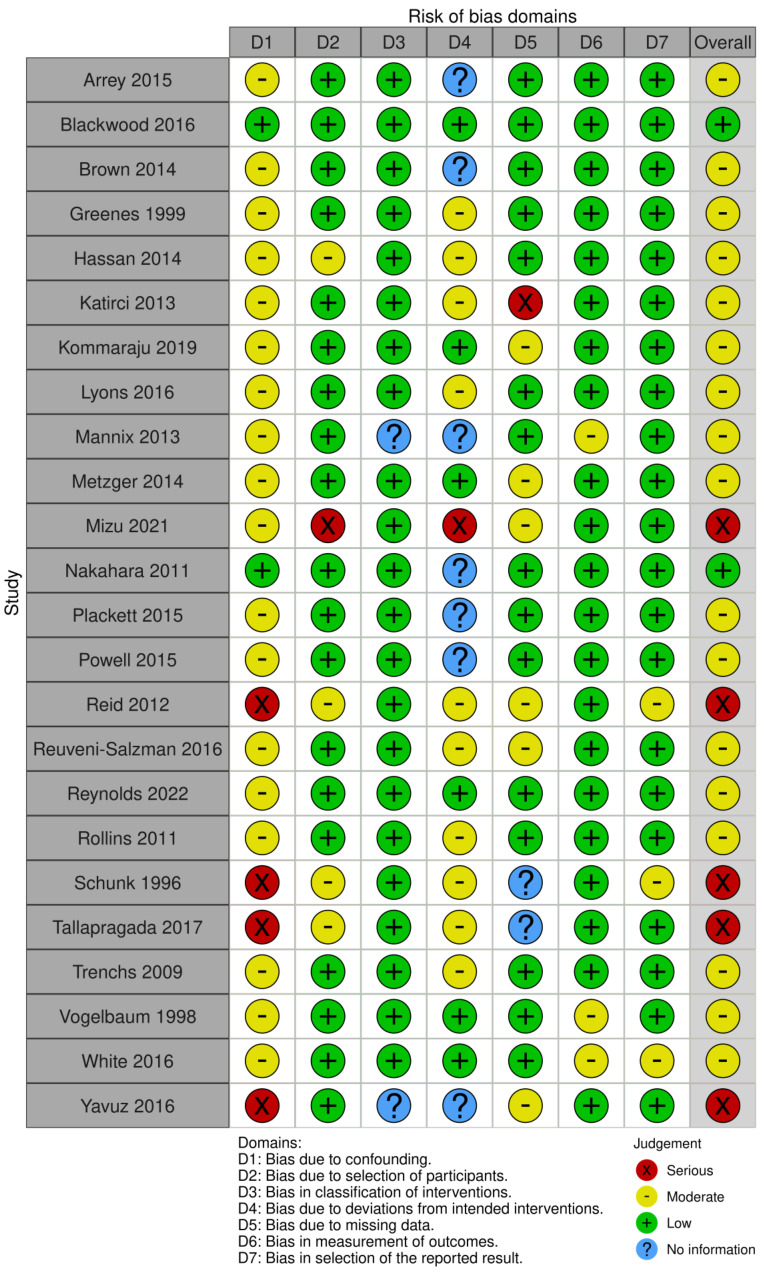
Quality assessment of the included studies employing the ROBINS-I scale [9,11,12,13,15,16,17,18,19,20,21,22,23,24,25,26,27,28,29,30,31,32,33,34].

Figure 2 uses a concise color-coded system based on the ROBINS-I scale to present the risk of bias among 25 studies. Green represents the two studies with “Low Risk of Bias”, yellow delineates the eighteen studies with “Moderate Risk of Bias”, and red highlights the five studies flagged for “Serious Risk of Bias”.

### 3.3. Patient Baseline Characteristics

A total of 10,350 skull fractures from 25 studies were included, of which 7228 were ISFs identified after CT scanning. Within the selected reports, 18 (72%) were retrospective analysis of patients’ characteristics and data. The United States of America (US) was the country in which most of the studies were established, encompassing 17 (68%) citations. From the included reports, twenty (80%) were initiated in this century; however, only five (20%) were conducted from 2010 onwards. Out of the total, four (16%) references were multicentric, with one being from Australia and the rest being from the US. One of these multicentric studies accounted for 44% of the included skull fractures. Except for Mannix et al. [20], all studies encompassed patients with equal or less than 18 years. Concerning the age of the patients, 16 (64%) studies included only patients with 15 years or less. When analyzing all of the reports, the initial Glasgow Coma Scale (GCS) score ranged from 13 to 15 in 2 (8%) and from 14 to 15 in 3 (12%) of them, whereas in 12 (48%) the GCS score was 15. A summary of the data can be examined in Table 1.

### 3.4. Outcomes from the Included Patients

In our sample, among the 7228 patients presenting ISFs confirmed by head CT, only two children underwent acute neurological surgery, representing virtually 0% of the patients. In contrast to the few patients who needed acute neurosurgery, a greater number of the children were hospitalized. In summary, 5351 (74%) of them were hospitalized. The length of their hospital stay did not exceed a period of time corresponding to 72 h, including an observation period and an eventual hospital admission. No deaths were reported in any of the 25 studies, despite the high number of hospitalized children and the two acute neurological procedures. Furthermore, only 10 studies provided useful data concerning the number of patients who underwent nonaccidental trauma evaluation. This amount, when reported, reached a total of 378 (5%) patients. Moreover, proceeding with the same rationale as Bressan et al. [6], the total number of patients that repeated neuroimaging varies in our whole study depending on the reported data of a single reference [33]. This reference does not specify the real number of patients with isolated nondisplaced linear skull fractures that repeated this imaging process, despite mentioning that 560 patients received a repeated CT. Thus, the data in this particular study is considered non reported. Hence, a total of 150 patients were identified as having repeated neuroimaging. Table 2 provides a greater view of the exposed data. Subsequently, a pooled analysis was performed.

### 3.5. Acute Neurosurgical Intervention

**Figure 3:**
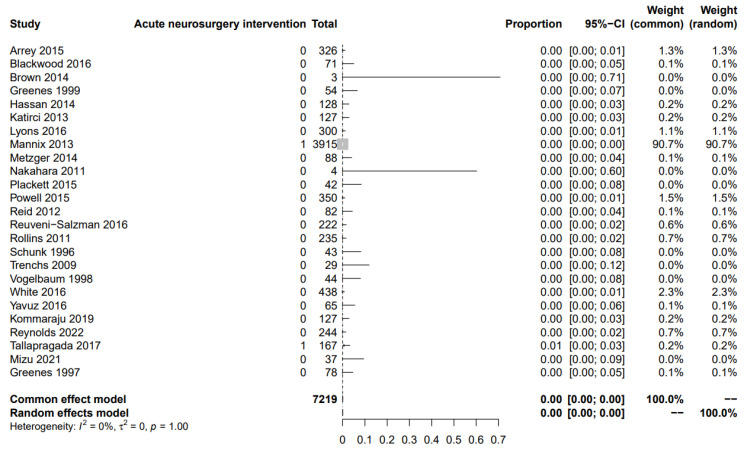
Patients who required acute neurosurgical intervention after an isolated skull fracture [9,11,12,13,14,15,16,17,18,19,20,21,22,23,24,25,26,27,28,29,30,31,32,33,34].

From 7219 patients from 25 studies, two required acute neurosurgical intervention (0,0%). After common and random analysis, the risk was calculated to be 0% (95% CI: 0–0%; I^2^ = 0%). The plot is available in Figure 3.

### 3.6. Hospitalization after Injury

**Figure 4:**
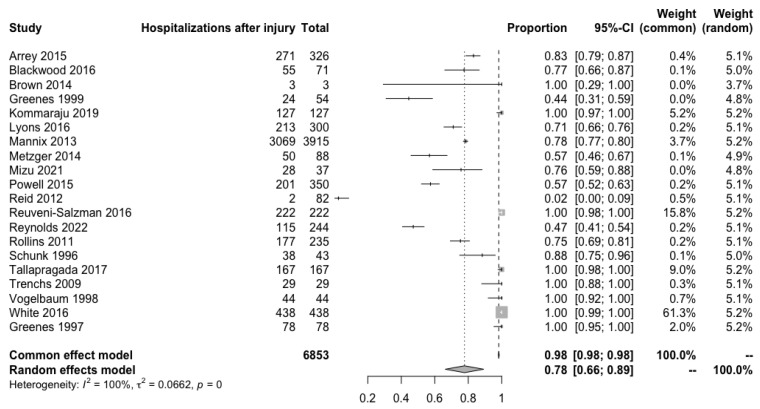
Patients who required hospitalization after an isolated skull fracture [9,11,12,13,14,15,18,19,20,21,22,25,26,27,28,29,30,31,32,33,34].

A sum of 6853 patients from 20 studies analyzed the incidence of hospitalization to more complete exams evaluation after the injury. Due to increased heterogeneity, after a random analysis, the risk of hospitalization was calculated to be 78% (95% CI: 66–89%; I^2^ = 100%). The most heterogeneous study was Reid et al. [26], in which only two patients out of eighty-two were hospitalized (2.4%). The statistics are depicted in Figure 4.

### 3.7. Repeated Neuroimaging

**Figure 5:**
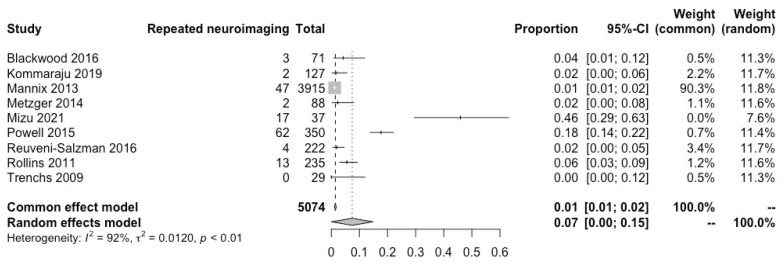
Patients who required repeated neuroimaging after an isolated skull fracture [12,18,20,21,22,25,27,28,31].

From nine studies with 5074 patients, neuroimaging was repeated in 150 patients (3%). Once again, due to high heterogeneity, after a random analysis the results came to a rate of 7% (95% CI: 0–15%; I^2^ = 92%). Mizu et al. [22] contributed significantly to the present heterogeneity. The outcome is illustrated in Figure 5.

## 4. Discussion

The management of ISFs in children is a multifaceted process that involves weighing the necessity of surgical intervention. Traditionally, these fractures have been perceived as potentially warranting surgery in severe cases to prevent or address complications such as epidural or subdural hematomas and other intracranial injuries [35]. In contrast, conservative management entails close observation, neuroimaging, and follow-up, and it is typically preferred for asymptomatic or mildly symptomatic isolated fractures. This approach aligns with the principle of minimizing invasive procedures in pediatric patients [36]. The positive outcomes observed in our meta-analysis, which support the efficacy of conservative management for ISFs, contribute significantly to the ongoing discourse on the most appropriate approach to these cases. These findings underscore the importance of a thorough evaluation to determine whether surgical intervention or medical hospitalization is genuinely necessary. While an isolated skull fracture typically bears a low positive predictive value for adverse outcomes, an escalation in risk emerges if supplementary information from the patient’s history, physical examination, laboratory results, additional imaging, or social work evaluation raises heightened concerns regarding the condition.

In our analysis, we highlight an exceptionally low incidence of acute neurosurgical intervention. Out of the 7219 patients analyzed, only two cases (0.0%) required such intervention, seen in Mannix et al. [20], and Tallapragada et al. [30] studies. This finding underscores the rarity of severe complications that necessitate surgical treatment in children with ISFs [37]. It suggests that a conservative approach, involving observation and non-operative management, is generally effective in managing these cases.

Because of these necessities, our study reports a notably high hospitalization rate among pediatric patients with ISFs. Approximately 78% of these patients were admitted to the hospital to receive a more complete evaluation of exams. The rationale behind this high hospitalization rate likely includes the need for close monitoring, repeated neurological assessments, and evaluation for potential complications, even though the incidence of surgical intervention is extremely low. While this cautious approach ensures the safety and thorough evaluation of these young patients, it also raises questions about the utilization of healthcare resources, the potential for over hospitalization in cases where conservative management may be more appropriate, when analyzing the amount spend annually for these scenarios [38], and the risk of iatrogenic damage caused by hospitalization itself [39].

Additionally, these discussions, advanced neuroimaging techniques play a pivotal role in assessing the extent and severity of injury, aiding in more informed decision-making, such as the PECARN Rule [40], developed to identify children at minimum risk of clinically significant TBI by diagnosing the extend and severity of the lesion. The ability to accurately diagnose and monitor these fractures using neuroimaging is an essential advancement [41]. The potential of emerging imaging technologies holds promise for future research and improving diagnostic accuracy [42]. Our study delves into the practice of repeating neuroimaging in pediatric patients with ISFs fractures, showing a routine repeat neuroimaging that does not consistently follow a homogeneous proportion across studies. The data suggests a repeat rate of 3%, which increases to 7% when accounting for high heterogeneity. This variation in practice reflects the lack of consensus on the necessity of repeat imaging, with some suggestions in the medical literature [43,44].

In summary, our analysis of the medical literature on pediatric skull fractures reveals a complex clinical landscape. Clinicians frequently recommend hospitalization for these cases at a notably high rate (78%), even though the ultimate need for neurosurgical intervention is relatively low. Moreover, there is substantial heterogeneity in the decision to repeat neuroimaging in our study. This variability may stem from unclear and non-uniform guidelines for managing such scenarios. We recognize the critical importance of decision-making in these situations and the absence of clear directives [45].

In light of these findings, data suggest a cautious approach among clinicians when contemplating surgical interventions and hospitalization. Emphasizing the significance of comprehensive clinical assessments and imaging becomes paramount in determining the appropriate course of action for each patient, and advocating to a conservative management unless there are clear clinical or neurological warning signs, and the skull imaging indicates no significant abnormalities.

## 5. Limitations

While our discussion has provided valuable insights into the management and outcomes of ISFs in pediatric patients, it is crucial to acknowledge several limitations that temper the interpretation of these findings.

The inherent heterogeneity in the data sources used for our meta-analysis is a notable concern. The included studies may have varied widely in terms of patient demographics, geographical locations, healthcare settings, and diagnostic criteria. This diversity can introduce variability in the results and may restrict the generalizability of our findings to a broader population of pediatric patients with ISFs.

Also, the potential for publication bias in meta-analyses cannot be overlooked. Studies with positive or statistically significant results are often more likely to be published than those with negative or non-significant findings. This bias could impact the overall outcomes of our analysis, potentially not fully representing the entirety of relevant research on this topic.

Another point of limitation is the quality of the included studies. Variability in study design, data collection methods, and reporting quality among the included studies could introduce biases or errors in our analysis. It is essential to recognize that the strength of our meta-analysis hinges on the quality of the underlying data.

Moreover, clinical heterogeneity among patients with ISFs is a complex issue that is difficult to fully account for in our analysis. Clinical factors such as the presence of associated injuries, neurological deficits, or other individual circumstances such as the pediatric population including more or less neonates may significantly influence the decision to pursue surgical intervention. These nuances may not be fully captured by the data we analyzed.

Furthermore, the temporal aspect must be considered. Our meta-analysis is based on data available up to a certain point in time. Clinical practices and guidelines can evolve over time, and new diagnostic technologies or approaches may have emerged since our data cutoff date, potentially impacting the management and outcomes of ISFs in children.

Ethical and cultural factors are also significant considerations. Our discussion does not delve into how these factors may influence clinical decision-making. Variations in healthcare practices and cultural norms can substantially impact whether surgical intervention is considered or favored in specific regions or healthcare settings.

Finally, the high rate of hospitalization that we observed in our analysis may not be universally applicable. Resource availability, healthcare infrastructure, and local practices can vary significantly across different regions and healthcare systems. The decision to hospitalize a child with an ISFs may be influenced by these contextual factors.

## 6. Conclusions

Our study delved into pediatric traumatic brain injuries, spotlighting ISFs. Out of 7228 children with such fractures confirmed via CT scans, an almost negligible number required immediate neurosurgical interventions, yet a significant 74% were hospitalized for up to 72 h. Notably, despite this high hospitalization rate, the mortality was zero, and repeat neuroimaging was uncommon.

This research is crucial in shedding light on the outcomes and implications of pediatric concerning ISFs. The apparent disparity between high hospitalization rates and the lack of severe outcomes necessitates further exploration—are these hospitalizations truly necessary? Future studies should prioritize understanding this dichotomy, employ rigorous randomized controlled trials, and assess the long-term impacts of these injuries, ultimately aiming to optimize treatment guidelines and observational protocols.

## Figures and Tables

**Table 1:** Baseline characteristics of included studies.

Study and Year	Study Design	Single or Multicenter	Enrolment Period	Local of Research	No. of Any Skull Fracture	No. of Isolated Skull Fracture (%)	Age Range	Initial GCS Score
Arrey 2015 [11]	R	Single	2009–2013	US	326	326 (100)	<15 years	NR
Blackwood 2016 [12]	R	Single	2004–2014	US	71	71 (100)	<12 years	15
Brown 2014 [13]	R	Single	2010–2011	UK	6	3 (50)	<1 year	NR
Greenes 1997 [14]	P	Single	1992–1994	US	105	78 (74)	<23 months	NR
Greenes 1999 [15]	P	Single	1998–1998	US	86	63 (74)	<2 years	NR
Hassan 2014 [16]	R	Single	2007–2010	US	223	128 (73)	<5 years	15
Katirci 2013 [17]	R	Single	2009–2010	Turkey	152	127 (84)	≤18 years	13–15
Kommaraju 2019 [18]	R	Single	2005–2015	US	127	127 (100)	≤18 years	14–15
Lyons 2016 [19]	P	Single	2008–2015	US	320	300 (94)	≤18 years	14–15
Mannix 2013 [20]	R	Multicenter	2005–2011	US	4596	3915 (85)	<19 years	NR
Metzger 2014 [21]	P	Single	2010–2014	US	88	88 (100)	≤16 years	15
Mizu 2021 [22]	R	Single	2011–2019	Japan	79	37 (47)	≤15 years	15
Nakahara 2011 [23]	R	Single	2005–2007	Japan	38	4 (11)	<4 years	15
Plackett 2015 [24]	R	Multicenter	2010–2014	US	42	42 (100)	≤13 years	15
Powell 2015 [25]	P	Multicenter	2004–2006	US	350	350 (100)	≤18 years	14–15
Reid 2012 [26]	R	Single	2003–2010	US	92	82 (89)	<2 years	15
Reuveni-Salzman 2016 [27]	R	Single	2006–2012	Israel	222	222 (100)	<14 years	15
Reynolds 2022 [9]	R	Single	2015–2017 and 2019–2020	US	244	244 (100)	≤18 years	NR
Rollins 2011 [28]	R	Single	2003–2008	US	1810	235 (13)	<14 years	15
Schunk 1996 [29]	R	Single	1992	US	79	43 (54)	<18 years	15
Tallapragada 2017 [30]	R	Multicenter	2009–2014	Australia	358	167 (47)	≤16 years	13–15
Trenchs 2009 [31]	P	Single	2004–2006	Spain	150	29 (19)	≤1 year	15
Vogelbaum 1998 [32]	R	Single	1993–1994	US	44	44 (100)	≤15 years	15
White 2016 [33]	R	Single	2005–2013	US	619	438 (71)	3.4 years (SD 4.1)	NR
Yavuz 2016 [34]	R	Single	1998–2000	Turkey	123	65 (53)	≤15 years	NR

Abbreviations: R—Retrospective; NR—Non reported, P—Prospecitve.

**Table 2:** Outcomes from the included patients.

Study	No. Isolated Skull Fracture on CT	No. Acute Neurosurgery (%)	No. Hospitalized (%)	Length of Hospital Stay	No. Deaths (%)	No. Nonaccidental Trauma Evaluation * (%)	No. Repeated Neuroimaging (%)
Arrey 2015 [11]	326	0	271 (83)	<72 h	0	24 (7)	NR
Blackwood 2016 [12]	71	0	55 (77)	<72 h	0	0	3 (4)
Brown 2014 [13]	3	0	3 (100)	<72 h	0	NR	NR
Greenes 1997 [14]	78	0	78 (100)	<72 h	0	NR *	NR
Greenes 1999 [15]	63	0	24 (44)	<72 h	0	NR	NR
Hassan 2014 [16]	128	0	NR	<72 h	0	NR	NR
Katirci 2013 [17]	127	0	NR	<72 h	0	NR	NR
Kommaraju 2019 [18]	127	0	127 (100)	<72 h	0	NR	2
Lyons 2016 [19]	300	0	213 (71)	<72 h	0	99 (31)	NR
Mannix 2013 [20]	3.915	1 (0.03)	3069 (78)	<72 h	0	186 (6)	47 (1)
Metzger 2014 [21]	88	0	50 (57)	<72 h	0	10 (23)	2 (2)
Mizu 2021 [22]	37	0	28 (76)	<72 h	0	NR	17 (46)
Nakahara 2011 [23]	4	0	NR	<72 h	0	NR	NR
Plackett 2015 [24]	42	0	NR	<72 h	0	NR	NR
Powell 2015 [25]	350	0	201 (57)	<72 h	0	NR	62 (18)
Reid 2012 [26]	82	0	2 (2)	<72 h	0	2 (2)	NR
Reuveni-Salzman 2016 [27]	222	0	222 (100)	<72 h	0	2 (1)	4 (2)
Reynolds 2022 [9]	244	0	115	<72 h	0	NR	NR
Rollins 2011 [28]	235	0	177 (75)	<72 h	0	2 (1)	13 (6)
Schunk 1996 [29]	43	0	38 (88)	<72 h	0	NR	NR
Tallapragada 2017 [30]	167	1 (0.6)	167 (100)	<72 h	0	NR	NR
Trenchs 2009 [31]	29	0	29 (100)	<72 h	0	NR	0
Vogelbaum 1998 [32]	44	0	44 (100)	<72 h	0	22 (50)	NR
White 2016 [33]	438	0	438 (100)	<72 h	0	31 (7)	NR **
Yavuz 2016 [34]	65	0	NR	<72 h	0	NR	NR

Abbreviations: NR—Non reported. * In ten studies, 101 patients with any skull fracture were evaluated child abuse, being found a total of thirty patients; number not specified for the subgroup of patients with linear nondisplaced skull fractures. ** In this study, 560 patients of the total 619 (181 with an isolated depressed skull fracture and 438 with an isolated nondisplaced skull fracture) received a repeated CT, and no children had new CT findings. The maximum and minimum number of patients with an isolated nondisplaced linear skull fracture who could have received a repeated CT ranged between 438 and 379.

## References

[1] Araki T., Yokota H., Morita A. (2017). Pediatric Traumatic Brain Injury: Characteristic Features, Diagnosis, and Management. Neurol. Med. Chir..

[2] Smith E.B., Lee J.K., Vavilala M.S., Lee S.A. (2019). Pediatric Traumatic Brain Injury and Associated Topics: An Overview of Abusive Head Trauma, Nonaccidental Trauma, and Sports Concussions. Anesthesiol. Clin..

[3] Ghajar J., Hariri R.J. (1992). Management of pediatric head injury. Pediatr. Clin. N. Am..

[4] Stark M.J., Hodyl N.A., Belegar V.K.K., Andersen C.C. (2016). Intrauterine inflammation, cerebral oxygen consumption and susceptibility to early brain injury in very preterm newborns. Arch. Dis. Child. Fetal Neonatal Ed..

[5] Ommaya A.K., Goldsmith W., Thibault L. (2002). Biomechanics and neuropathology of adult and paediatric head injury. Br. J. Neurosurg..

[6] Bressan S., Marchetto L., Lyons T.W., Monuteaux M.C., Freedman S.B., Da Dalt L., Nigrovic L.E. (2018). Systematic Review and Meta-Analysis of the Management and Outcomes of Isolated Skull Fractures in Children. Ann. Emerg. Med..

[7] Kochanek P.M., Tasker R.C., Bell M.J., Adelson P.D., Carney N., Vavilala M.S., Selden N.R., Bratton S.L., Grant G.A., Kissoon N. (2019). Management of Pediatric Severe Traumatic Brain Injury: 2019 Consensus and Guidelines-Based Algorithm for First and Second Tier Therapies. Pediatr. Crit. Care Med..

[8] Kochanek P.M., Tasker R.C., Carney N., Totten A.M., Adelson P.D., Selden N.R., Davis-O’reilly C., Hart E.L., Bell M.J., Bratton S.L. (2019). Guidelines for the Management of Pediatric Severe Traumatic Brain Injury, Third Edition: Update of the Brain Trauma Foundation Guidelines, Executive Summary. Neurosurgery.

[9] Reynolds R.A., Kelly K.A., Ahluwalia R., Zhao S., Vance E.H., Lovvorn H.N., Hanson H., Shannon C.N., Bonfield C.M. (2022). Protocolized management of isolated linear skull fractures at a level 1 pediatric trauma center. J. Neurosurg. Pediatr..

[10] Page M.J., McKenzie J.E., Bossuyt P.M., Boutron I., Hoffmann T.C., Mulrow C.D. (2021). The PRISMA 2020 statement: An updated guideline for reporting systematic reviews. BMJ.

[11] Arrey E.N., Kerr M.L., Fletcher S., Cox Jr C.S., Sandberg D.I. (2015). Linear nondisplaced skull fractures in children: Who should be observed or admitted?. J. Neurosurg. Pediatr..

[12] Blackwood B.P., Bean J.F., Sadecki-Lund C., Helenowski I.B., Kabre R., Hunter C.J. (2016). Observation for isolated traumatic skull fractures in the pediatric population: Unnecessary and costly. J. Pediatr. Surg..

[13] Brown C.W., Akbar S.P., Cooper J.G. (2014). Things that go bump in the day or night: The aetiology of infant head injuries presenting to a Scottish Paediatric Emergency Department. Eur. J. Emerg. Med..

[14] Greenes D.S., Schutzman S.A. (1997). Infants with isolated skull fracture: What are their clinical characteristics, and do they require hospitalization?. Ann. Emerg. Med..

[15] Greenes D.S., Schutzman S.A. (1999). Clinical indicators of intracranial injury in head-injured infants. Pediatrics.

[16] Hassan S.F., Cohn S.M., Admire J., Nunez-Cantu O., Arar Y., Myers J.G., Dent D.L., Eastridge B.J., Cestero R.F., Gunst M. (2014). Natural history and clinical implications of nondepressed skull fracture in young children. J. Trauma Acute Care Surg..

[17] Katirce Y., Ocak T., Karamercan M.A., Kocaşaban D., Yurdakul M.S., Başpınar I., Coşkun F. (2013). Compliance with Catch Rules in Administering Computerized Tomography Scans to Children Admitted to the Emergency Department with Minor Head Trauma. Acta Med. Mediterr..

[18] Kommaraju K., Haynes J.H., Ritter A.M. (2019). Evaluating the Role of a Neurosurgery Consultation in Management of Pediatric Isolated Linear Skull Fractures. Pediatr. Neurosurg..

[19] Lyons T.W., Stack A.M., Monuteaux M.C., Parver S.L., Gordon C.R., Gordon C.D., Proctor M.R., Nigrovic L.E. (2016). A QI Initiative to Reduce Hospitalization for Children with Isolated Skull Fractures. Pediatrics.

[20] Mannix R., Monuteaux M.C., Schutzman S.A., Meehan W.P., Nigrovic L.E., Neuman M.I. (2013). Isolated skull fractures: Trends in management in US pediatric emergency departments. Ann. Emerg. Med..

[21] Metzger R.R., Smith J., Wells M., Eldridge L., Holsti M., Scaife E.R., Barnhart D.C., Rollins M.D. (2014). Impact of newly adopted guidelines for management of children with isolated skull fracture. J. Pediatr. Surg..

[22] Mizu D., Matsuoka Y., Huh J.Y., Onishi M., Ariyoshi K. (2021). Head CT findings and deterioration risk in children with head injuries and Glasgow Coma Scales of 15. Am. J. Emerg. Med..

[23] Nakahara K., Shimizu S., Utsuki S., Oka H., Kitahara T., Kan S., Fujii K. (2011). Linear fractures occult on skull radiographs: A pitfall at radiological screening for mild head injury. J. Trauma.

[24] Plackett T.P., Asturias S., Tadlock M., Wright F., Ton-That H., Demetriades D., Esposito T., Inaba K. (2015). Re-evaluating the need for hospital admission and observation of pediatric traumatic brain injury after a normal head CT. J. Pediatr. Surg..

[25] Powell E.C., Atabaki S.M., Wootton-Gorges S., Wisner D., Mahajan P., Glass T. (2015). Isolated linear skull fractures in children with blunt head trauma. Pediatrics.

[26] Reid S.R., Liu M., Ortega H.W. (2012). Nondepressed linear skull fractures in children younger than 2 years: Is computed tomography always necessary?. Clin. Pediatr..

[27] Reuveni-Salzman A., Rosenthal G., Poznanski O., Shoshan Y., Benifla M. (2016). Evaluation of the necessity of hospitalization in children with an isolated linear skull fracture (ISF). Child’s Nerv. Syst..

[28] Rollins M.D., Barnhart D.C., Greenberg R.A., Scaife E.R., Holsti M., Meyers R.L., Mundorff M.B., Metzger R.R. (2011). Neurologically intact children with an isolated skull fracture may be safely discharged after brief observation. J. Pediatr. Surg..

[29] Schunk J.E., Rodgerson J.D., Woodward G.A. (1996). The utility of head computed tomographic scanning in pediatric patients with normal neurologic examination in the emergency department. Pediatr. Emerg. Care.

[30] Tallapragada K., Peddada R.S., Dexter M. (2018). Paediatric mild head injury: Is routine admission to a tertiary trauma hospital necessary?. ANZ J. Surg..

[31] Trenchs V., Curcoy A.I., Castillo M., Badosa J., Luaces C., Pou J., Navarro R. (2009). Minor head trauma and linear skull fracture in infants: Cranial ultrasound or computed tomography?. Eur. J. Emerg. Med..

[32] Vogelbaum M.A., Kaufman B.A., Park T.S., Winthrop A.L. (1998). Management of uncomplicated skull fractures in children: Is hospital admission necessary?. Pediatr. Neurosurg..

[33] White I.K., Pestereva E., Shaikh K.A., Fulkerson D.H. (2016). Transfer of children with isolated linear skull fractures: Is it worth the cost?. J. Neurosurg. Pediatr..

[34] Yavuz M.S., Asirdizer M., Cetin G., Günay Balci Y., Altinkok M. (2003). The correlation between skull fractures and intracranial lesions due to traffic accidents. Am. J. Forensic Med. Pathol..

[35] Teasdale G.M., Murray G., Anderson E., Mendelow A.D., MacMillan R., Jennett B., Brookes M. (1990). Risks of acute traumatic intracranial haematoma in children and adults: Implications for managing head injuries. BMJ.

[36] Lerwick J.L. (2016). Minimizing pediatric healthcare-induced anxiety and trauma. World J. Clin. Pediatr..

[37] Hassan S., Alarhayema A.Q., Cohn S.M., Wiersch J.C., Price M.R. (2018). Natural History of Isolated Skull Fractures in Children. Cureus.

[38] Schutzman S.A., Greenes D.S. (2001). Pediatric minor head trauma. Ann. Emerg. Med..

[39] Walsh K.E., Landrigan C.P., Adams W.G., Vinci R.J., Chessare J.B., Cooper M.R., Hebert P.M., Schainker E.G., McLaughlin T.J., Bauchner H. (2008). Effect of computer order entry on prevention of serious medication errors in hospitalized children. Pediatrics.

[40] Gambacorta A., Moro M., Curatola A., Brancato F., Covino M., Chiaretti A., Gatto A. (2022). PECARN Rule in diagnostic process of pediatric patients with minor head trauma in emergency department. Eur. J. Pediatr..

[41] Mulroy M.H., Loyd A.M., Frush D.P., Verla T.G., Myers B.S., Bass C.R. (2012). Evaluation of pediatric skull fracture imaging techniques. Forensic Sci. Int..

[42] Jeong T.S., Yee G.T., Kim K.G., Kim Y.J., Lee S.G., Kim W.K. (2023). Automatically Diagnosing Skull Fractures Using an Object Detection Method and Deep Learning Algorithm in Plain Radiography Images. J. Korean Neurosurg. Soc..

[43] Chateil J.F. (2011). Head trauma in children—How to image?. Pediatr. Radiol..

[44] O’Brien Sr W.T., Caré M.M., Leach J.L. (2018). Pediatric Emergencies: Imaging of Pediatric Head Trauma. Semin. Ultrasound CT MR..

[45] Easter J.S., Bakes K., Dhaliwal J., Miller M., Caruso E., Haukoos J.S. (2014). Comparison of PECARN, CATCH, and CHALICE rules for children with minor head injury: A prospective cohort study. Ann. Emerg. Med..

